# Correlation of neuroendocrine features with prognosis of non-small cell lung cancer

**DOI:** 10.18632/oncotarget.12327

**Published:** 2016-09-28

**Authors:** Jianguo Feng, Huaying Sheng, Chihong Zhu, Xiaoqian Qian, Danying Wan, Dan Su, Xufeng Chen, Liming Zhu

**Affiliations:** ^1^ Key Laboratory Diagnosis and Treatment Technology on Thoracic Oncology, Hangzhou, Zhejiang 310022, China; ^2^ Cancer Research Institute, Zhejiang Cancer Hospital, Hangzhou, Zhejiang 310022, China; ^3^ Department of Thoracic Oncology, Zhejiang Cancer Hospital, Hangzhou, Zhejiang 310022, China; ^4^ Department of Pathology and Laboratory Medicine, David Geffen School of Medicine at UCLA, Los Angeles, California 90095, USA; ^5^ Department of Chemotherapy, Zhejiang Cancer Hospital, Hangzhou, Zhejiang 310022, China

**Keywords:** non-small cell lung cancer, neuroendocrine, CD56, CgA, Syn

## Abstract

The improvement in histological diagnostic tools, including neuroendocrine markers by immunohistochemistry (IHC), has led to increased recognition of non-small cell lung cancer (NSCLC) with neuroendocrine (NE) feature. However, little is known regarding the prevalence and clinical implications of NE feature in patients with NSCLC. In this study, we performed IHC in a tissue microarray containing 451 Chinese NSCLC cases, and analyzed correlation of the expression of neuroendocrine marker with pathological and clinical features of NSCLC. The result showed that NE feature in NSCLC was detectable in almost 30% of studied patients, and tumors with NE feature were significantly correlated with pathological classification, clinical stages and cell differentiation of NSCLC. Our data also revealed that NE feature indicated worse overall survival and disease free survival. Compared with mutant p53, NE markers showed more significance as for prognostic evaluation. Multi-factor COX analysis further suggested a potential clinical impact for NE feature as an independent indicator of poor prognosis for NSCLC patients.

## INTRODUCTION

Lung cancer remains one of the leading causes of cancer mortality worldwide [1, 2]. In China, the morbidity and mortality of lung cancer has been rising due to smoking and environmental deterioration in the past decades [3–6]. Despite the tremendous efforts and progress in lung cancer research, and the use of aggressive multimodal chemo- and radiotherapy, the overall treatment outcome for lung cancer patients remains poor.

Primary carcinomas of the lung are traditionally classified as either small cell lung cancer (SCLC) or non-small cell lung cancer (NSCLC). NSCLC constitutes approximately 85% of all primary lung cancers with adenocarcinoma, squamous cell carcinoma (SCC) and large cell carcinoma constituting the major histological types [7]. NSCLC are often diagnosed at late stages [8]. For patients with advanced stages of NSCLC when surgical excision is not an option, the adjuvant chemotherapy and radiotherapy has been extensively used [9]. However, unlike small-cell lung cancer (SCLC) that often has neuroendocrine (NE) features, NSCLC is usually chemoresistant. Of interest, retrospective studies indicated that a subgroup of NSCLC patients with NE features may benefit from chemotherapeutic treatment. Thus, it is extremely important to be able to clarify this subgroup of NSCLC, and determine the clinical impacts of NE feature in NSCLC patients.

NE feature has been detected in 10%–20% of histologically ordinary NSCLCs. These include large-cell neuroendocrine carcinoma (LCNEC, 3%), NSCLC with uncertain neuroendocrine differentiation (NSCLC-ND, 3%) and composite lung cancer with neuroendocrine tumor cells (5%) [10–12]. However, little is known regarding the prevalence and clinical implications of NE feature in patients with NSCLC.

In this study, we evaluated the potential prognostic values of NE feature in NSCLC. We performed immunohistochemical assays for NE markers CD56, synaptophysin (SYN) and chromogranin A (CgA), and tumor suppressor p53 in tissue microarray containing 451 cases of NSCLC. Retrospective analyses showed that expressions of CD56, SYN and CgA were significantly correlated to pathological classification, tumor differentiation, and clinical (TNM) stages. Kaplan-Meier curve analysis also indicated that NSCLC patients with tumors of NE feature had worse overall survival and disease free survival. Compared to mutant P53, NE markers had more significance for prognostic evaluation. Multi-factor COX analysis further showed that NE feature was the independent risk factors of poor prognosis for NSCLC patients.

## RESULTS

### Expressions of CD56, CgA, SYN and mutant p53 in NSCLC

**Figure 1:**
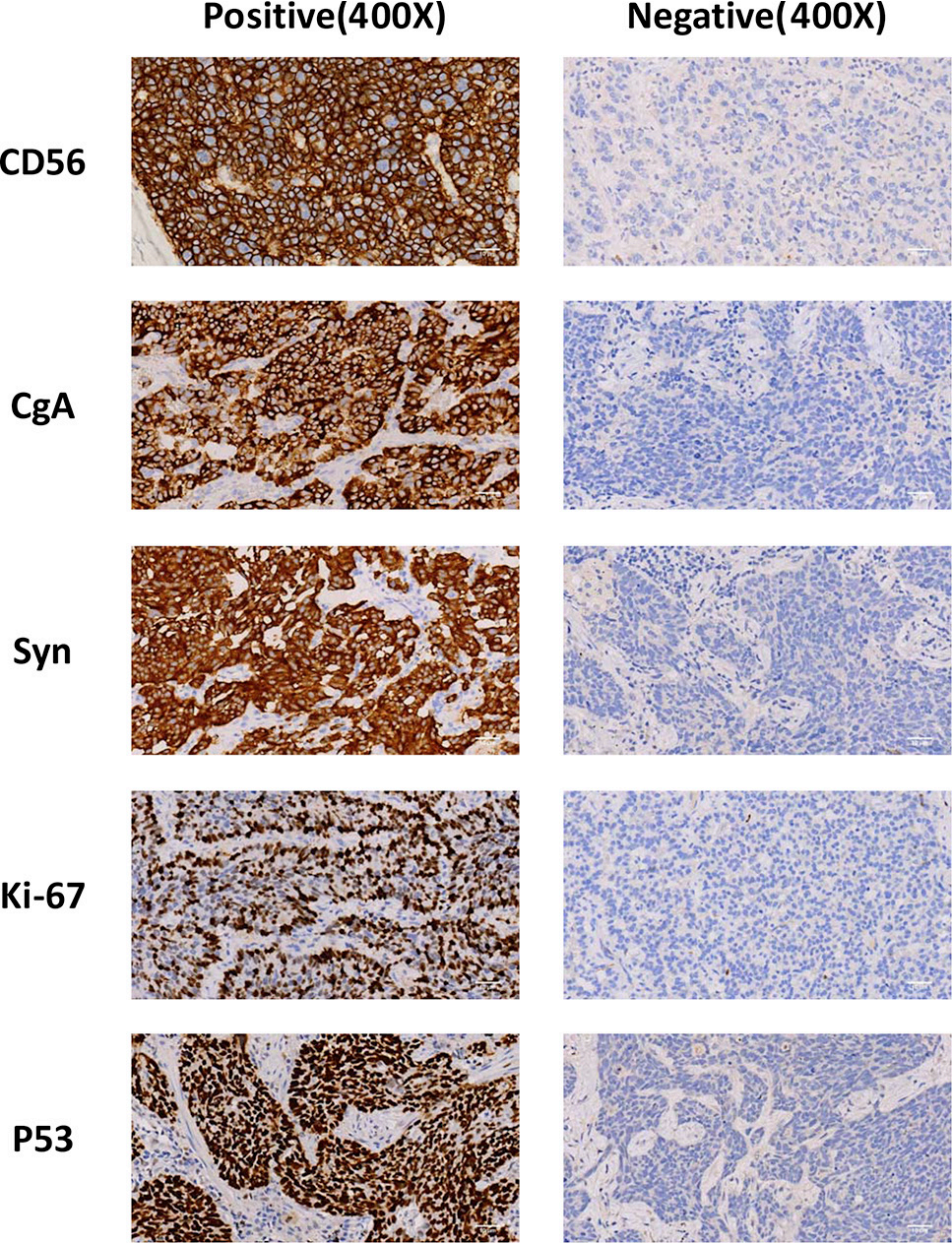
Expression of CD56, CgA, Syn and mutatn p53 in NSCLC Samples were stained by immunohistochemical method, and representative images of positive and negative staining were shown as indicated. Images were taken under microscope and magnified by 400 × fold.

In this tissue microarray, we observed positive immunostaining for CD56, CgA and SYN in 60 (13.3%), 134 (29.7%) and 86 (19.1%) cases, respectively (Figure 1, Table 1 and Supplementary Figure S1). Of them, 85 (18.85% of 451 cases) specimen were stained with two or more indicated NE markers, and these cases were then considered as tumors with neuroendocrine feature (NE feature). Because mutation of the p53 gene is one of the most significant molecular events occurring in about 50% of NSCLC and plays important roles in the tumorigenesis of lung epithelial cells and resistance to clinical treatments [13], we also included IHC for p53 in this study and the positive immunostainings of mutant p53 was detected in 312 (69.2%) cases. Of interest, Chi-squared analyses indicated that there were significant correlations of the expressions of these three NE markers or NE feature with that of mutant p53 (*p* values all < 0.001).

**Table 1:** Correlation of CgA, CD56, Syn and NE feature with mutant p53

		P53		*P*
		Negative (*n*)	Positive (*n*)	
CgA	Negative	114	203	< 0.001
	Positive	25	109	
CD56	Negative	132	259	< 0.001
	Positive	7	53	
Syn	Negative	123	242	< 0.01
	Positive	16	70	
NE	Negative	124	242	< 0.01
	Positive	15	70	

### Pathological implications of NE marker and NE feature for NSCLC

The potential diagnostic values of these molecular markers were then analyzed. As shown in Table 2, we found that adenocarcinoma had higher percentages of tumors expressing each individual NE marker, or mutant p53, or with NE feature than squamous carcinoma (*p* all < 0.001). Compared to middle-high grade of tumors, tumors in low-middle grade also showed higher rates, with statistical significances, for expressions of three NE markers or with NE feature (*p* all < 0.05). However, no such association was observed with expression of mutant p53. We further found that the expressions of CD56 or SYN, or tumor with NE feature, were associated with TNM staging of NSCLC (*p* all < 0.05), and tumors at later staging had higher percentages of expressions for these molecular markers. Of interest, we did not observed correlation between CgA expression and TNM staging. In addition, we found no associations for expressions of NE markers, or NE feature, with other pathological factors such as gender, age and family history (*p* > 0.05). Of note, a correlation of CgA expression was found to be associated with smoking (*p* = 0.042). These results indicated that expression of NE markers or tumors with NE feature are associated with histological type, tumor grade or differentiation, and TNM staging for NSCLC.

**Table 2:** Correlations of NEmarkers and NE features with clinicopathlogical parameters

Factors		CD56		*P*	CgA		*P*	Syn		*P*	NE		*P*	P53		*P*
		−	+		−	+		−	+		−	+		−	+	
		*n*	*n*		*n*	*n*		*n*	*n*		*n*	*n*		*n*	*n*	
Sex	Male	294	46	0.805	247	93	0.055	278	62	0.43	279	61	0.389	107	223	0.601
	Female	97	14		70	41		87	24		87	24		32	79	
Age	< 65	273	40	0.622	221	96	0.823	250	63	0.389	258	55	0.297	99	214	0.575
	≥ 65	118	20		92	42		115	23		108	30		40	98	
Family History	No	312	48	0.97	254	106	0.71	292	68	0.774	294	66	0.516	249	111	0.928
	Yes	77	12		61	28		71	18		70	19		62	27	
Smoking	Never	128	16	0.348	92	52	0.042	114	30	0.514	115	29	0.631	41	103	0.46
	Ever/Current	263	44		225	82		251	56		251	56		98	209	
Alcohol	Never	199	28	0.505	157	70	0.452	184	43	0.818	188	39	0.533	66	161	0.365
	Ever/Current	189	32		160	61		181	40		178	43		73	148	
Histologic type	Squamous cell carcinoma	209	17	< 0.001	188	38	< 0.001	197	29	0.001	204	22	< 0.001	80	146	0.027
	Adenocarcinoma	174	41		123	92		159	56		154	61		54	161	
	Others*	8	2		6	4		9	1		8	2		5	5	
Grade	High-middle	202	20	0.008	182	42	< 0.001	189	33	0.017	194	28	0.001	70	152	0.658
	Middle-low-low	184	39		132	91		170	53		167	56		66	157	
Clinical stage	I	168	15	< 0.001	139	44	0.082	153	30	0.017	163	20	< 0.001	57	126	0.854
	II	97	14		77	34		87	24		88	23		36	75	
	III	126	31		102	55		125	32		115	42		46	111	

* Otherhistologic type includes alveolar cancer, neuroendocrine cancer, giant cell carcinoma, carcinosarcoma, pleomorphic carcinoma and pulmonary blastoma and others.

### Tumor with expressions of NE markers were correlated with poor prognosis of NSCLC

**Figure 2:**
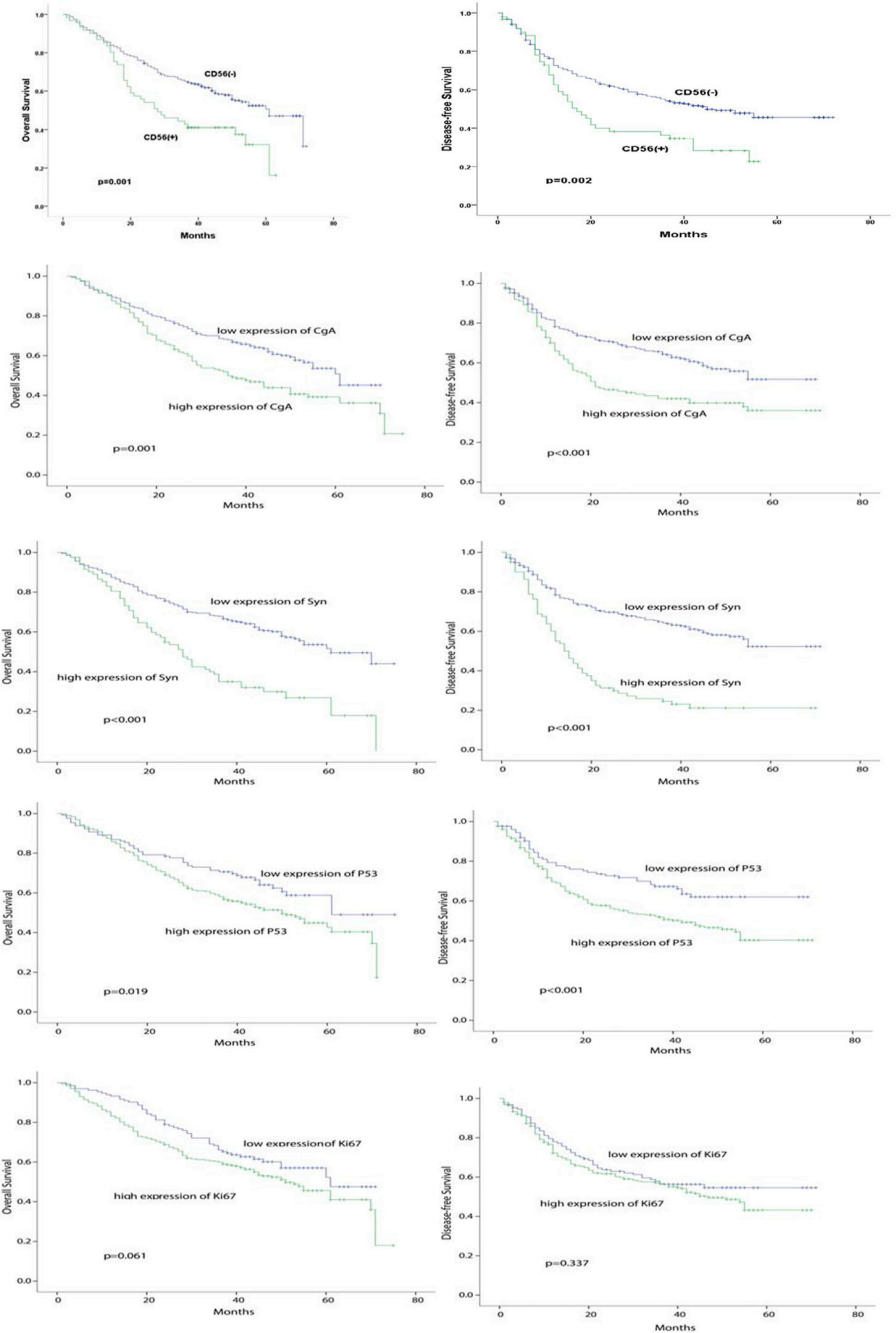
Correlation of CD56, CgA, Syn, NE feature and mutant p53 with prognosis of NSCLC Correlation of CD56, CgA, Syn, NE feature and mutant p53 with DFS and OS of NSCLC patients were analyzed by kaplan-meier method using Log rank statistics. *p* value < 0.05 indicating statistical significance.

Kaplan-Meier analysis and Log-rank test showed that the expressions of NE markers (CD56, CgA, SYN) or mutant p53 were associated with overall survival (OS) and disease free survival (DFS). Our data present in Figure 2 showed that higher expression of these markers indicated OS worse and DFS; of them, expression of SYN had the most significant values for both DFS and OS (*p* < 0.001).

**Figure 3:**
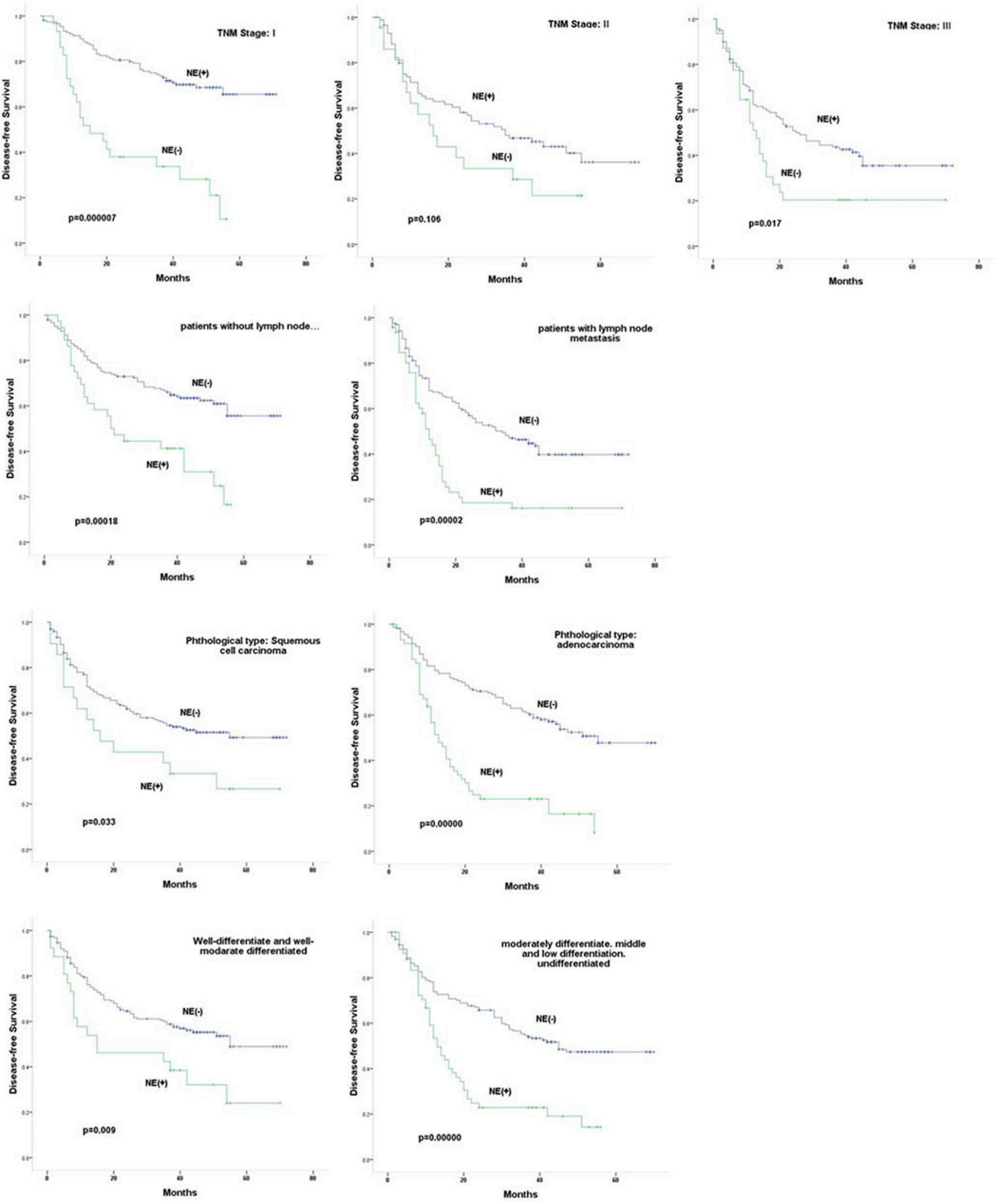

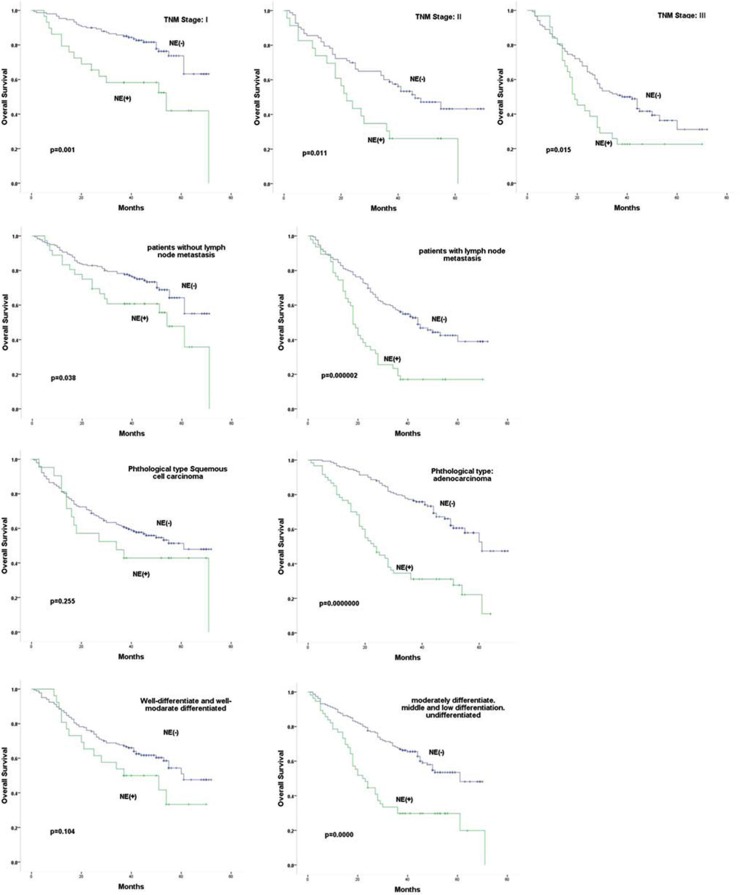
Correlation of NE feature with prognosis of NSCLC Correlation of NE feature with DFS and OS of NSCLC patients at same staging, in same group of patients with or without lymph-node metastasis, in same group of patients with adenocarcinoma or with squamous carcinoma, or in same group of patients with different grade of tumors were analyzed by kaplan-meier method using Log rank statistics. *p* value < 0.05 indicating statistical significance.

We further evaluated potential prognostic values of NE feature in NSCLC patients at same staging or in same groups of patients with or without lymph-node metastasis. As shown in Figure 3, our results revealed that tumors with NE feature indicated worse DFS or OS with statistical significances for patients at same TNM staging of I and III/IV (*p* all < 0.005); however, such correlation was only observed for OS (*p* = 0.011) but not for DFS in stage II patients (*p* = 0.106). NE feature also indicated worse DFS and OS for patients diagnosed with lymph-node metastasis (*p* both < 0.001). In patients that no lymph-node metastasis was found, however, NE feature only indicated worse DFS (*p* < 0.001), but had no prognostic value indicating worse OS (*p* = 0.038). NE feature also showed prognostic value predicting both worse DFS and OS (*p* both < 0.001) for patients with adenocarcinoma. For patients with squamous carcinoma, NE feature only indicated worse DFS (*p* = 0.033), but not OS (*p* = 0.255). In addition, tumors with NE feature were also associated with worse DFS and OS (*p* both < 0.001) for patients with un differentiated or low-middle grade of tumors, and were associated DFS (*p* = 0.009) but not OS (*p* = 0.104) for patients with middle-high grade tumors.

Taken together, these results suggested potential prognostic values of the NE markers, or NE feature, for NSCLC patients.

### NE feature is an independent risk factor for prognosis

The potential prognostic values of NE feature, p53 and clinical characteristics of patients were further analyzed by multi-factor COX analysis. Results showed that NE feature and TNM stage were significantly correlated to DFS (*p* < 0.001 for NE feature and *p* = 0.004 TNM staging) and OS (*p* = 0.006 for NE feature and *p* < 0.001 for TNM staging), and expression of mutant p53 was correlated to DFS (*p* = 0.025) but not OS (*p* = 0.16) for NSCLC patients in Kaplan-Meier curve; however, COX regression analysis indicated that only NE feature had most significance as an independent risk factor for poor prognosis (Table 3).

**Table 3:** Multivariate analysis of clinicopathological characteristics with prognosis

Variable	DFS			OS		
	HR	95% CI	*P* Value	HR	95% CI	*P*Value
NE	1.73	1.28–2.33	0.00001	1.52	1.13–2.05	0.006
P53	1.50	1.05–2.14	0.025	1.28	0.91–1.79	0.160
Gender	0.86	0.51–1.44	0.567	0.85	0.49–1.45	0.543
Age	1.19	0.879–1.62	0.258	1.28	0.94–1.73	0.112
GradeGroup	1.13	0.84–1.52	0.417	1.36	0.85–1.52	0.396
Histotype	1.16	0.94–1.43	0.158	0.94	0.76–1.16	0.544
StageGroup	1.54	1.15–2.07	0.004	2.06	1.55–2.74	0.000
Family_History	1.07	0.76–1.51	0.695	1.04	0.74–1.47	0.809
Smoking	1.22	0.77–1.94	0.401	1.29	0.80–2.06	0.304
Drinking	0.923	0.667–1.28	0.567	0.94	0.69–1.28	0.690

## DISCUSSION

Neuroendocrine carcinoma represent 25% of primary lung neoplasms, including a spectrum of tumors from the low-grade typical carcinoid (TC) and intermediate-grade atypical carcinoid (AC) to the high-grade SCLC and large-cell NE carcinoma (LCNEC) [14]. There tumors are characterized by the expression of panendocrine markers, neuroamines, and neuropeptides and by ultrastructural evidence of dense-core secretory granules. Recent studies using morphologic analyses, immunohistochemical studies and molecular studies have attempted to conceptualize neoplasms with NE features to provide a better understanding in terms of clinical course, natural behavior, and possible histogenesis.

The treatment approaches to lung neuroendocrine carcinomas are markedly different: carcinoid tumors are primarily treated with surgical resection and SCLC is generally considered as a nonsurgical disease; there is no consensus on the clinical management of LCNEC, as the efforts to establish treatment guidelines for LCNEC are hampered by the relative rarity of this tumor and the challenges in diagnostic reproducibility [15, 16]. LCNEC are considered as an aggressive form of NSCLC and may also be sensitive to chemotherapy [17–22], 5-year actuarial survival for LCNEC patients with surgical resction ranges from 13% to 57% [21, 23, 24]. Of note, lung neuroendocrine carcinomas have higher regional lymph node metastasis and distant metastasis (even in patients with low-grade of TC) than generally-termed NSCLC [16], which may account for, at least partially, the poor clinical outcomes of these patients.

Approximately 10–20% of overall NSCLC was found to show NE differentiation. Studies have attempted to conceptualize this family of NSCLC to provide a better understanding in terms of clinical course, natural behavior and possible histogenesis, however, the results were controversial in terms of histological entities and clinical implications [25, 26].

In this study, we performed immunohistochemistry for NE markers CD56, SYN and CgA, and for tumor suppressor p53 in a tissue microarray containing 471 cases of NSCLC. Our result showed that NE feature was detectable in almost 30% of studied patients. Chi-square analysis demonstrated that NE feature was apparently associated with histological type of adenocarcinoma, low-middle grade tumors, clinical staging, DFS of NSCLC. Although OS for patients can be affected by the different treatment plans for patients after surgical resection, we found that tumors with NE feature were obviously associated with worse OS for patients. Our data also indicated that NE feature is a significant and independent risk factor for poor prognosis of NSCLC patients.

Because of that mutant p53 is closely related to cancer cell proliferation and tumor progression [27, 28], we also analyzed the correlation of individual NE marker or NE feature with expressions of mutant P53, and the results demonstrated that these NE markers or NE feature were significantly associated with the expression of mutant P53.

In conclusion, the results from this present study supported that NE feature is not uncommon in NSCLC, and NE feature in NSCLC is correlated to clinical parameters such as histological type, clinical staging and tumor grade. Although previous studies showed controversial results for NE feature, or NE differentiation, as predicator for prognosis of clinical therapy [29, 30], our results present here revealed significant prognostic values of NE feature for NSCLC patients. These results suggest potential clinical impacts for NE feature as a diagnostic tool for NSCLC and an independent predictor for prognosis of NSCLC patients, and may also provide essential insights for optimization of clinical treatments for NSCLC patients. However, further analyses including whether patients with NSCLC of NE feature may have different responses to chemotherapy or radiotherapy are needed.

## MATERIALS AND METHODS

### Clinical sample

451 cases of primary tumor tissues were collected from surgical specimens of NSCLC patients during a period from March 2008 to April 2010. Tissue samples were paraffin-embedded, and tissue microarray was then prepared according to Hematoxylin & Eosin (H.E) staining results. Procedures for collection of these tumor tissues and for preparation of tissue microarray were approved by the Medical Ethics Committee of Zhejiang cancer Hospital.

All specimens were reviewed histologically by independent pathologists, according to WHO histological classification of lung cancer [31] and Union for International Cancer Control (UICC) TNM-staging in 2010 [32]. Of these 451 cases, there were 217 cases of adenocarcinoma, 227 cases of squamous carcinoma and 10 cases of others (including alveolar carcinoma, neuroendocrine carcinoma, large cell carcinoma, pleomorphic carcinoma, basaloid carcinoma, epithelioma-like carcinoma, sarcomatoid carcinoma, mucoepidermoid carcinoma, invasive carcinoma, etc.); 183 cases were diagnosed of stage I (34 cases of stage Ia and 149 cases of stage Ib), 110 cases of stage II (1 case of stage IIa and 110 cases of stage IIb) and 157 cases of stage III (115 cases of stage IIIa and 42 cases of stage IIIb). There were 340 males and 111 females, aging from 30 to 82 with an average age of 60.4 ± 8.7. Of them, forty-seven patients had received preoperative radiotherapy or chemotherapy or other treatments before surgery.

All of the patients were followed through telephone communications with themselves or relatives until Jan. 30, 2013. Recurrence or metastasis was found in 226 patients, and 200 patients of them died during the follow-up period; there were 14 cases without the data; 18 cases (4.0%) were lost to follow-up during survival time, and 13 cases (2.9%) were lost to follow-up during disease progression, including death cases but without data of metastasis.

### Tissue microarray

The paraffin-embedded slices were chosen and the needed locations of paraffin-embedded tissue were selected and marked according to H.E staining results. Blank receptor paraffin box was made by tissue embedder and paraffin-embedded pathological tissue cores with 1 mm in diameter and 3 mm in depth were taken out and arranged in the blank receptor paraffin box regularly using a tissue arranger according to the designed histological type and arrangement for the study. The tissue blocks were then heated in an oven under 52°C for fusion to make the tissue microarray and receptor paraffin block combined together closely. The paraffin blocks were adjusted and sliced for H.E staining and subsequent histochemical assay.

### Immunohistochemistry

Rabbit polyclonal anti-CgA and anti-SYN were purchased from Abcam (Cambridge, MA, US) and were used with dilution of 1:80 for IHC. Rabbit polyclonal anti-p53 (DO-7) was from Fuzhou Maixin Biotech (Fujian, China) with dilution of 1:100 for IHC. Mouse monoclonal anti CD56 was from DAKO with dilution of 1:110 for IHC. Immunostaining was performed using a standard avidin-biotin-peroxidase complex method according to the manufacturers' instructions (AURAGENE, Changsha, China). Briefly, slices were deparaffinized in xylene and rehydrated in graded alcohol baths. Antigen retrieval was performed by boiling the sections in 0.01 M citrate buffer for 2 min in an autoclave. Hydrogen peroxide was used to block endogenous peroxide activity and the normal goat serum was used to reduce nonspecific binding. After preparation, slices were incubated at the room temperature for one hour with the primary antibodies with optimized dilutions. Mouse or rabbit immunoglobulin were used as negative controls. After incubation with the biotinylated secondary antibody, the slices were washed with PBS and then incubated with streptavidin–biotin conjugated with horseradish peroxidase. Freshly prepared DAB chromogenic reagent was added dropwise on the slices to develop color and then rinsed by running water to terminate the color developing and re-stained by hematoxylin and using PBS to go back to blue.

### Evaluation and scoring of immunostaining

Two independent pathologists randomly reviewed and scored each stained tissue section under a 400-fold magnification for semi-quantitative assessment as previously reported [33, 15]. In brief, 5 fields with 100 cells for each field were evaluated per slide, and at least 100 cells were evaluated per field. Score of staining intensity of tumor cell ranged from 0 to 3 (0: no staining, 1: weak staining, 2: moderate staining, 3: strong staining), and score of percentage of positive cells ranged from 0 to 3 (0: < 5%, 1: 5%–25%, 2: 25%–50%, 3: > 50%); staining intensity and percentage of positive cells were observed and calculated by integration using the following formula: (+)% ×1+(++)% ×2+(+++)% ×3. It would be (+) when the integration below 1.0, (++) when 1.0–1.5 and (+++) when above 1.5.

### Statistical analysis

The correlation of clinical data with the expressions of each molecular marker was studied by multiple regression analysis, chi-squared analysis and COX regression analysis; differential expression of protein, survival and disease progression were studied by Kaplan-Meier log rank test. *P* < 0.05 was considered as statistically significant difference.

### The novelty and impact statement

The clinical implication of neuroendocrine differentiation in NSCLC remains unclear. With immunohistochemstry on a tissue microarray containing 451 cases of non-small cell lung cancer (NSCLC), we showed here that neuroendocrine differentiation is not uncommon in NSCLC, and expressions of neuroendocrine biomarkers are correlated to tumor differentiation of grade and TNM staging. Our finding that neuroendocrine differentiation in NSCLC tumors indicates poorer survival may provide important clinical impact for NSCLC patients.

## SUPPLEMENTARY MATERIALS FIGURE


