# Supplementary material for: Correlation of neuroendocrine features with prognosis of non-small cell lung cancer
Source: Oncotarget. 2016 Sep 28;7(44):71727–36. doi: 10.18632/oncotarget.12327 (PMC5342116; doi:10.18632/oncotarget.12327)
Supplement: Supplementary file 1 [file oncotarget-07-71727-s001.pdf]

Correlation of neuroendocrine features with prognosis of non-small cell lung cancer

Supplementary Materials

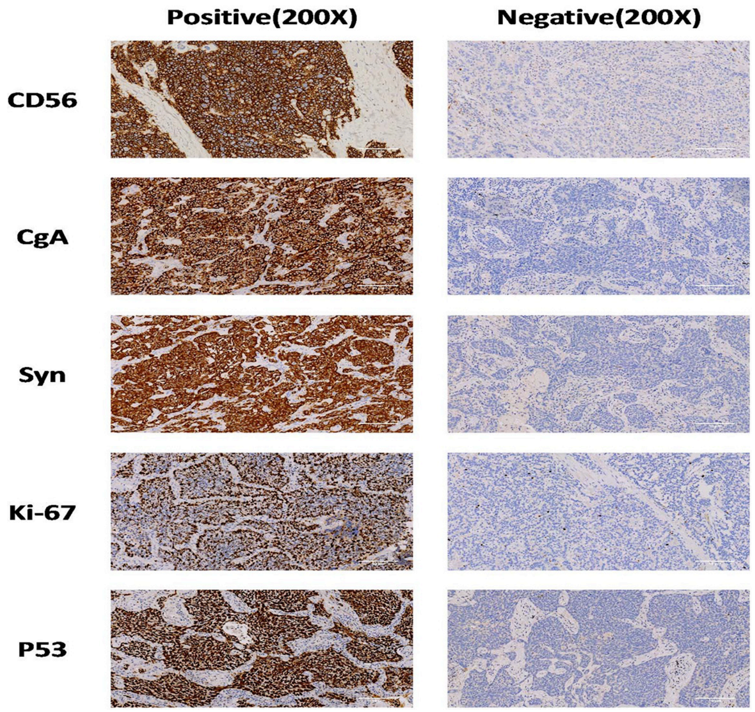

Supplemetary Figure S1: Samples were stained by immunohistochemical method, and representative images of positive and negative staining were shown as indicated. Images were taken under microscope and magnified by 200× fold.
